# Expression Patterns of DNA Methylation and Demethylation Genes during Plant Development and in Response to Phytohormones

**DOI:** 10.3390/ijms22189681

**Published:** 2021-09-07

**Authors:** Morgan Bennett, Kailyn Cleaves, Tarek Hewezi

**Affiliations:** Department of Plant Sciences, University of Tennessee, Knoxville, TN 37996, USA; mmbennet@vols.utk.edu (M.B.); kcleave1@vols.utk.edu (K.C.)

**Keywords:** Arabidopsis, DNA demethylases, DNA methyltransferases, embryogenesis, GUS activity, plant growth and development, phytohormones

## Abstract

DNA methylation and demethylation precisely and effectively modulate gene expression during plant growth and development and in response to stress. However, expression profiles of genes involved in DNA methylation and demethylation during plant development and their responses to phytohormone treatments remain largely unknown. We characterized the spatiotemporal expression patterns of genes involved in de novo methylation, methyl maintenance, and active demethylation in roots, shoots, and reproductive organs using β-glucuronidase (GUS) reporter lines. Promoters of DNA demethylases were generally more highly active at the mature root tissues, whereas the promoters of genes involved in DNA methylation were more highly active at fast-growing root tissues. The promoter activity also implies that methylation status in shoot apex, leaf primordia, floral organs, and developing embryos is under tight equilibrium through the activity of genes involved in DNA methylation and demethylation. The promoter activity of DNA methylation and demethylation-related genes in response to various phytohormone treatments revealed that phytohormones can alter DNA methylation status in specific and redundant ways. Overall, our results illustrate that DNA methylation and demethylation pathways act synergistically and antagonistically in various tissues and in response to phytohormone treatments and point to the existence of hormone-linked methylome regulation mechanisms that may contribute to tissue differentiation and development.

## 1. Introduction

Epigenetic mechanisms coordinate the expression of thousands of genes in a heritable yet flexible manner, without changing the underlying DNA sequence [1]. DNA methylation, along with histone modifications, chromatin remodeling, and non-coding small RNAs (sRNAs) constitute the highly dynamic and interconnected epigenome, which contributes to the regulation of gene expression during plant development and stress response [2,3,4,5,6,7,8,9]. DNA methylation is also involved in transposable element (TE) regulation, reproduction, genomic imprinting, intercellular communication, interallelic communication, and genome interactions [5]. DNA methylation is generally regarded as a repressive mark of gene expression. DNA methylation in the promoter of protein coding and microRNA genes has been generally shown to cause transcriptional repression, although recent reports point to a possible role of DNA methylation in gene transcriptional activation [3,10,11,12,13]. The functional role of DNA methylation in gene body regions, however, remains largely unknown even though recent studies associate DNA methylation levels and patterns with gene expression and splicing efficiency [14,15,16].

DNA methylation is the addition of a methyl group to the C5 position of cytosine and occurs in the CG, CHG, and CHH sequence contexts (where H is any nucleotide except G) [17]. In Arabidopsis, the de novo formation of 5-methylcytosine (5mC) is catalyzed by DNA methyltransferases domains rearranged methyltransferase (DRM1), DRM2, and DRM3 through the RNA-directed DNA methylation (RdDM) pathway, with DRM2 being the key enzyme in this pathway [18,19,20].

DNA methylation is maintained by distinct sequence context-dependent enzymes following DNA replication and cell division [21]. Methylation in the CG context is maintained by methyltransferase 1 (MET1) [22]. There is also evidence that MET1 paralogs MET2b and MET3 contribute to the maintenance of CG methylation [23]. Variant in methylation1 (VIM1), VIM2, and VIM3 and the heterochromatin-remodeling component decrease in DNA methylation (DDM1) are also considered important factors for CG DNA methylation [24,25,26]. CHG methylation is maintained by chromomethylase2 (CMT2) and CMT3 [27]. Kryptonite (KYP/SUVH4) and the histone methyltransferase SUVH5 and SUVH6 also contribute to the maintenance of CHG sites [27,28]. For CHH methylation, CMT2 maintains DNA methylation within pericentromeric long TEs, whereas the RdDM pathway is employed for the maintenance of CHH methylation at other loci [24,29]. The catalytically mutated paralog DRM3 is required for de novo methylation and maintenance of methylated CHH loci at repetitive sequences [20,30]. In addition, CMT1 and DRM1/2 also participate in the maintenance of non-CG methylation [27,28,29].

As with any balanced system, active DNA demethylation is an equally important epigenetic process for controlling genome-wide methylation levels [31,32,33,34,35]. In Arabidopsis, four DNA demethylase paralogs have been identified for active DNA demethylation: repressor of silencing1 (ROS1), demeter (DME), DME-like2 (DML2) and DML3 [31,33,36,37,38,39]. These DNA demethylases remove methylated cytosines through the base excision repair (BER) mechanism and replace them with an unmethylated cytosine [40]. Experimental evidences indicate that DNA methylation and active DNA demethylation are highly synchronized [41,42]. ROS1 is tightly controlled by the methylation level of an RdDM target sequence upstream of the transcriptional start site, called a DNA methylation monitoring sequence (MEMS). MEMS is hypermethylated when cellular RdDM increases or DNA demethylation decreases. MEMS methylation upregulates ROS1, thereby activating DNA demethylation [41].

DNA methylation-mediated transcriptional reprogramming has been reported in various plant developmental processes, from gametogenesis to fruit ripening. Local and global DNA methylation and demethylation are also heavily involved in plant responses to environmental stimuli, including biotic [7,43,44,45] and abiotic [8,9,46,47,48] stresses. However, the nature of the biochemical signals that associate with DNA methylome changes during plant development and stress responses are not understood. Phytohormone signaling pathways are involved in almost all aspects of plant growth, development, and stress responses [49,50,51,52]. Because phytohormones, similar to epigenetic modifications, are regulated in a special and temporal manner and have the ability to provide plants with regulatory mechanisms to quickly adapt to their biotic and abiotic environments, several studies point to a role for phytohormone signaling in controlling epigenetic pathways [53,54,55,56]. However, direct experimental evidence showing the functional relationship between phytohormones and epigenetic modifications remains lacking.

Many orthologous DNA methyltransferases and demethylases have been identified in non-model plant species as well as evolutionary unrelated organisms including invertebrates, humans, and other mammals. Thus, DNA methylation pathways are thought to be conserved and functionally significant. DNA methylation has long been recognized as a critical component of gene regulation, biochemical and hormonal balance, and environmental plasticity [45,57,58,59]. The expression patterns of individual genes involved in DNA methylation in specific tissues or organs have been previously reported [60,61,62]. However, comparative analyses of the spatial and temporal expression patterns of key DNA methylation and demethylation-related genes throughout plant growth and development remain missing. In this study, we determined the promoter activity of 15 genes involved in DNA methylation and active DNA demethylation in roots, shoots, and reproductive tissues using GUS reporter lines. In order to better understand the connection between DNA methylation and phytohormones, we also assessed the spatiotemporal expression patterns of genes involved in DNA methylation and demethylation in roots and shoots in response to treatment of six phytohormones: auxin (AUX), cytokinin (CK), gibberellic acid (GA), ethylene (ET), abscisic acid (ABA), and salicylic acid (SA). Our results reveal a unique and overlapping expression patterns of these genes in roots, shoots, inflorescences, siliques, and developing seeds, and highlight the importance of DNA methylation–demethylation equilibrium in these tissues. Additionally, our results point to a role of phytohormone signaling in establishing plant methylomes associated with the differentiation and development of various tissues.

## 2. Results

### 2.1. Generation of GUS Reporter Lines of 15 DNA Methylation and Demethylation-Related Genes

In order to assess the spatial and temporal expression patterns of genes involved in DNA methylation and demethylation in various plant tissues and organs, a number of transgenic GUS reporter lines were generated. The 15 selected genes include known cytosine methyltransferases, DNA demethylases (or glycosylases), and other genes required for DNA methylation, all of which are listed in Supplementary Table S1. Each reporter construct expresses GUS under the control of the gene’s respective native promoter (i.e., up to approximately 2 kb upstream of the translation start codon). GUS expression assays were performed and analyzed using at least two independent transgenic lines per construct in two-week-old roots, shoots, and in various reproductive tissues and organs.

### 2.2. Promoter Activity of DNA Methylation and Demethylation-Related Genes in Roots

**Figure 1:**
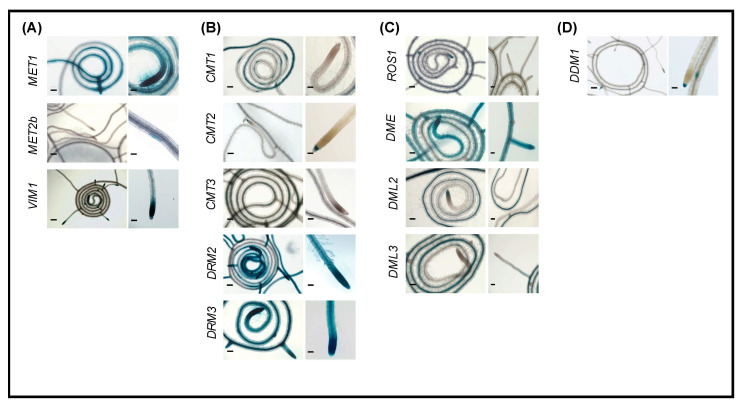
Promoter activity of DNA methylation and demethylation-related genes in two-week-old roots. A–C: Histochemical localization of GUS activity directed by promoters of the CG methylation-related genes *MET1*, *MET2b*, and *VIM1* (**A**), the non-CG and RdDM-related methyltransferases *CMT1*, *CMT2*, *CMT3*, *DRM2*, and *DRM3* (**B**), the demethylases *ROS1*, *DME*, *DML2*, and *DML3* (**C**), and *DDM1* (**D**). Scale bar = 5 mm.

Several genes involved in DNA methylation, including *MET1*, *CMT1*, *DRM2*, and *DRM3,* are highly expressed throughout root tissues (Figure 1). The CG methyltransferase *MET1* is highly expressed toward the newly grown parts of the root and into the root tips as well as in developing lateral roots (Figure 1A). However, other genes involved in CG methylation, *MET2b*, *MET3*, and *VIM2* have very limited expression in roots (Figure 1A and Supplementary Figure S1A). *VIM1* expression is observed in the tips of main and lateral roots and in the vascular tissues of the newly grown parts of the root (Figure 1A). 

Promoter of the de novo methyltransferases *DRM2* and *DRM3* are strongly active in most of the root tissues, particularly in the newly grown parts of the root and in the root tip, similarly to *MET1* (Figure 1B). Thus, it seems likely that DRM2 and DRM3 participate in de novo CG methylation, and MET1 maintains those methylated cytosines following DNA replication in rapidly diving root tissues. While *CMT1* is expressed in the majority of root tissues, its expression is mostly towards the mature end of the roots (i.e., towards the shoot) as well as at the base of the lateral roots (Figure 1B). Similar to *CMT1*, *CMT3* is expressed at the mature end of the root, but to a lesser extent (Figure 1B). Therefore, it appears that CMT1 and CMT3 have more specific roles in non-CG methyl maintenance at the mature end of the root tissues. The DNA demethylase *ROS1* is expressed exclusively in the root apical meristem (RAM) and in the root caps of the main and auxiliary roots (Figure 1C). *DME*, *DML2*, and *DML3*, on the other hand, are expressed throughout the root, with *DME* being the most highly expressed (Figure 1C). *DML2* and *DML3* have similar expression patterns, predominantly towards the mature end. These data support the conclusion that DME, DML2, and DML3 have overlapping functions in active DNA demethylation in most parts of the root tissues. Of note is that *DDM1*, which is required for the maintenance of CG and non-CG methylation [28], is uniquely expressed in the root cap of primary root and lateral root primordia (Figure 1D).

### 2.3. Promoter Activity of DNA Methylation and Demethylation-Related Genes in Shoots

**Figure 2:**
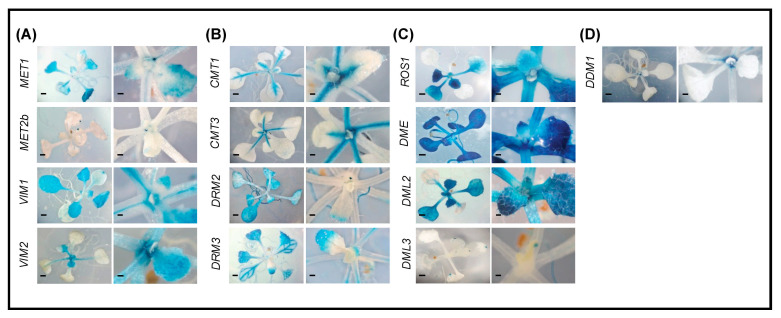
Promoter activity of DNA methylation and demethylation-related genes in two-week-old shoots. A–C: Histochemical localization of GUS activity directed by the promoters of the CG methylation-related genes *MET1*, *MET2b*, *VIM1*, and *VIM2* (**A**), the non-CG and RdDM-related methyltransferases *CMT1*, *CMT3*, *DRM2*, and *DRM3* (**B**), the demethylases *ROS1*, *DME*, *DML2*, and *DML3* (**C**), and *DDM1* (**D**). Scale bar = 5 mm.

GUS staining shows that all tested DNA methyltransferases and demethylases are expressed in the two-week-old shoot tissues (Figure 2). Interestingly, all these genes are transcriptionally active in leaf primordia, suggesting a role for DNA methylation-demethylation equilibrium in leaf morphogenesis. Additionally, the promoter of *MET1*, *MET2b*, *VIM2*, and *DDM1* showed activity in the shoot apex (Figure 2A,D). Of the genes involved in DNA methylation, *MET1*, *VIM1*, *VIM2*, *CMT1*, *DRM2*, and *DRM3* were highly expressed in leaf tissues (Figure 2A,B). *VIM1* and *VIM2* showed stage-specific expression patterns in leaves, where *VIM1* is expressed in mature leaves and *VIM2* is expressed in younger tissues (Figure 2A). *CMT1* and *DRM2* showed distinct expression in leaves. *CMT1* is highly expressed in leaf petiole and vascular tissues, whereas *DRM2* is expressed in leaf blades but not in the petioles or leaf vascular tissue (Figure 2B). To the contrary, *MET1* and *DRM3* display expression patterns similar to each other. Both are expressed in leaf blades and in cotyledon vascular tissues (Figure 2A,B), suggesting a coordinated methylation of CG and CHH loci in these tissues.

Notably, *ROS1*, *DME*, and *DML2* appear to be the most highly active genes in leaf primordia (Figure 2C), suggesting that DNA demethylation plays an important role during the early stages of leaf differentiation and development. The DNA demethylases *ROS1*, *DME*, and *DML2* are highly expressed in shoots (Figure 2C). *ROS1* and *DML2* are strongly expressed, particularly in young leaves. *DME*, on the other hand, is highly expressed in leaves, cotyledons, and trichomes, irrespective of leaf developmental stage. These data suggest that DME contribute to active DNA demethylation throughout leaf and cotyledon development and that ROS1 and DML2 contribute to this process only during the early stages of leaf development. The localized expression patterns of *DML3* at the tips of leaves and cotyledons suggest its contribution to active DNA demethylation, specifically in these regions.

### 2.4. Promoter Activity of DNA Methylation and Demethylation in Reproductive Tissues

#### 2.4.1. Buds and Open Flowers 

**Figure 3:**
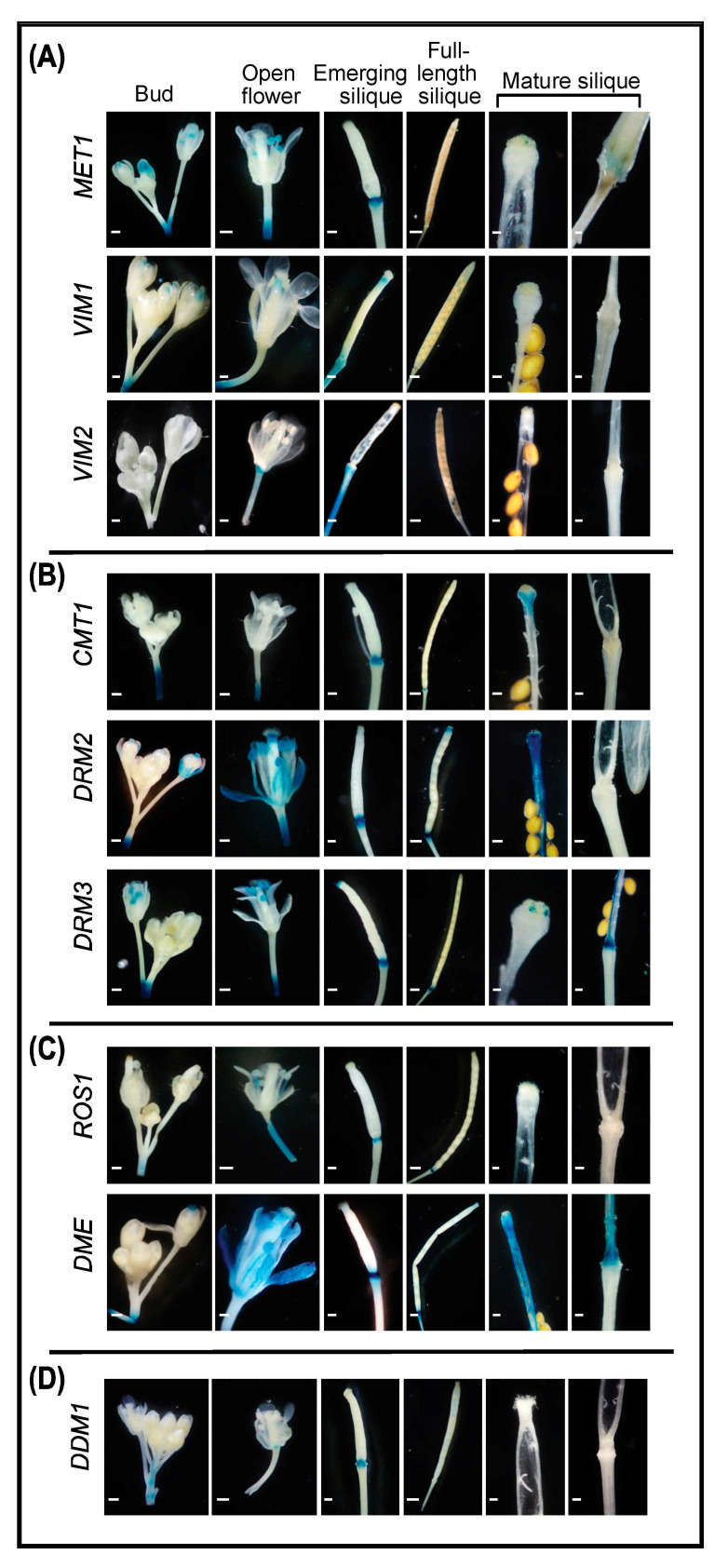
Promoter activity of DNA methylation and demethylation-related genes in floral tissues and organs. A–C: Histochemical localization of GUS activity directed by promoters of the CG methylation-related genes *MET1*, *VIM1*, and *VIM2* (**A**), the non-CG and RdDM-related *CMT1*, *DRM2*, and *DRM3* methyltransferases (**B**), and the demethylases *ROS1* and *DME* (**C**), and *DDM1* (**D**) in inflorescence buds, open flowers, emerging siliques, distal end of mature siliques, and proximal end of mature siliques. Scale bar = 5 mm.

GUS activity reveals that several genes involved in DNA methylation and demethylation are expressed in open flowers and buds (Figure 3A,B). Of the tested genes, only the CG methyltransferase *MET1* was expressed in unopened floral buds. *MET1*, *VIM1*, *DRM2*, *DRM3*, *DME*, and *ROS1* are expressed in various tissues of open flowers. Remarkably, *DRM2* and *DME* showed strong expression in petals, anthers, and stigma, suggesting a role for these two enzymes in DNA methylation fine-tuning during flower development (Figure 3B,C). *ROS1* is expressed in filaments, suggesting a unique role for DNA demethylation in this structure (Figure 3C). In addition to *MET1*, *DRM2*, *DRM3*, and *DME* are highly expressed in anthers (Figure 3A–C), reinforcing the hypothesis that DRM2 functions in parallel with DRM3 in RdDM pathways [20,30,63]. Taken together, these results demonstrate that DNA methylation and demethylation pathways are active during flowering and fertilization.

#### 2.4.2. Siliques

DNA methylation and demethylation pathways are particularly active in the junction between the silique and the pedicel (Figure 3). This region is composed of the gynophore, nectaries, and abscission zone and functions in attaching the silique to the pedicel until mature seeds are ready for dispersal [64]. In the emerging siliques, *VIM1* promoter is active in the pedicel, internode junction, and style (Figure 3A). *VIM2* expression is predominant in the pedicel, but distinctly absent from the internode junction (Figure 3A). Based on these results, VIM1 could be involved in the early stages of fruit ripening and pedicel development in association with VIM2. 

It was also evident from GUS activity that the genes involved in CG methylation are mostly expressed in early stages of silique development, whereas the genes involved in de novo methylation, non-CG methylation, and DNA demethylation were expressed throughout silique development (Figure 3). Notably, *DRM2* and *DMR3* appear to have roles in DNA methylation of the distal and proximal ends of mature siliques, respectively. In addition, *CMT1* and *DRM2* show overlapping expression patterns in the style of mature siliques (Figure 3B). *DME* is the highest DNA demethylase expressed in mature siliques (Figure 3C).

#### 2.4.3. Seeds

**Figure 4:**
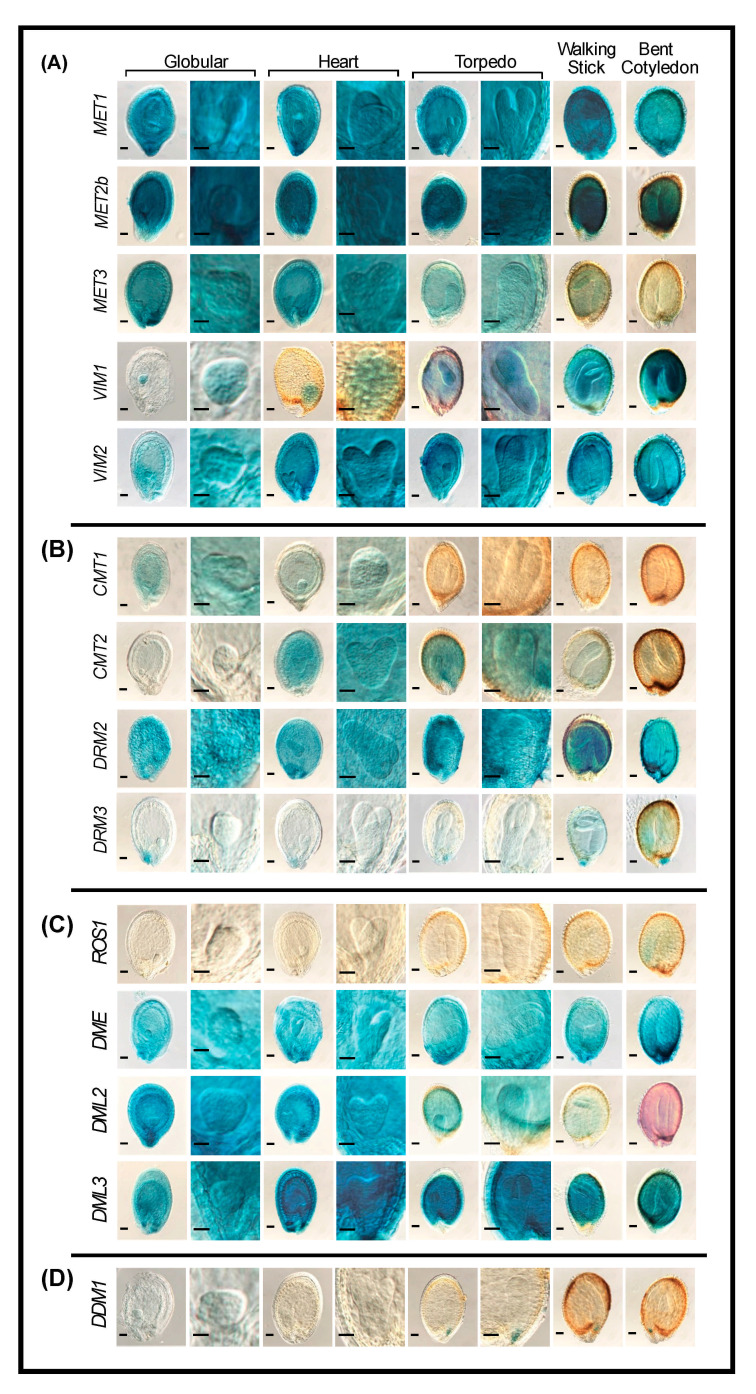
Promoter activity of DNA methylation and demethylation-related genes during various stages of seed development. A–C: Histochemical localization of GUS activity directed by promoters of the CG methylation-related genes *MET1*, *MET2b*, *MET3*, *VIM1*, and *VIM2* (**A**), the non-CG and RdDM-related methyltransferases *CMT1*, *CMT2*, *DRM2*, and *DRM3* (**B**), the demethylases *ROS1, DME*, *DML2*, and *DML3* (**C**), and *DDM1* (**D**) at globular, heart, torpedo, walking-stick, and bent-cotyledon embryo stages. Scale bar = 5 mm.

Our results demonstrate that promoters of many genes involved in DNA methylation and demethylation are also active in developing seeds from the globular to mature stages of embryogenesis. The CG methyltransferases *MET1* and *MET2b* are highly expressed, both in the endosperm and embryo throughout all stages of embryogenesis, whereas *MET3* is highly expressed during early embryo developmental stages and decreases with advancement in development (Figure 4A). In contrast to *MET3*, *VIM2* showed increased expression in endosperm and embryo with advancement in development (Figure 4A). *VIM1* is expressed exclusively at the globular and heart stages of embryogenesis. However, during the walking-stick and bent-cotyledons embryo stages, the expression of *VIM1* was observed both in endosperm and embryonic tissues (Figure 4A). 

*DRM2* showed high expression, both in endosperm and embryo throughout all embryo developmental stages (Figure 4B), reflecting its role in de novo DNA methylation in all sequence contexts via the RdDM pathway and in maintenance of asymmetric CHH methylation through persistent de novo methylation during various stages of seed development. *CMT1* and *CMT2* seem to be expressed in a sequential manner during embryogenesis. While *CMT1* is expressed at the globular and heart stages, *CMT2* is expressed at the heart, torpedo, and walking-stick stages (Figure 4B). *CMT3* was the only gene without detectable expression during embryogenesis under our experimental conditions (Supplementary Figure S1D). *DRM3* exhibits specific expression in the elaiosome at all embryo stages. Weak expression of *DRM3* was also detected in the embryo and endosperm at the walking-stick and bent-cotyledon stages (Figure 4B). 

The DNA demethylases *DME* and *DML3* exhibit strong GUS staining in endosperm and embryo during all embryo developmental stages (Figure 4C). High activity of *DML2* promoter was observed during early stages of embryogenesis (globular and heart), and then declined in more advanced embryonic stages (torpedo and walking-stick) (Figure 4C). *ROS1* showed the lowest expression among the four DNA demethylases, as its expression was barely detected only at the bent-cotyledon stage (Figure 4C). *DDM1* is specifically expressed at the tip of the radical in later stages of embryo development (Figure 4D). Together, these results suggest that embryogenesis and seed development involve dynamic reprogramming of DNA methylation and active demethylation. 

### 2.5. RNA-seq and RT-qPCR Data Support Promoter Activity of DNA Methylation and Demethylation-Related Genes

To provide confirmation of our promoter activity data, the gene expression levels of DNA methylation and demethylation-related genes in the root, root tips, leaf, petiole, bud, open flowers, and mature silique of wild-type plants were retrieved from the TraVA RNA-seq database [65] and compared with GUS activity. The gene expression levels are generally in agreement with the observed GUS activity of DNA methylation and demethylation-related genes in various tissues. For example, high expression levels of *MET1*, *DRM3*, and *DME* in root and root tips are in agreement with our data, showing strong GUS activity of these three genes in these tissues (Supplementary Figure S2). Similarly, the high expression levels of *ROS1* and *DME* in leaf and leaf petiole, *MET1, VIM1, DRM2, DRM3*, and DME, and in buds and open flower, and DME in mature silique, are consistent with GUS activity (Supplementary Figure S2). Additionally, the relative low GUS activity of *MET2b*, *MET3*, *CMT1*, and *DML3* in various tissues are congruent with RNA-seq data (Supplementary Figure S2). 

To further validate the GUS activity, we used reverse transcription quantitative PCR (RT-qPCR) to quantify the expression of *VIM2*, *CMT3*, and *DRM3* in the root tissues of two-week-old wild-type Col-0 plants. *VIM2* showed the lowest expression level, followed by a higher expression of *CMT3* (3.75 fold) and *DRM3* (7.51 fold) (Supplementary Figure S3A). These expression data are consistent with GUS activity of *VIM2*, *CMT3*, and *DRM3*, which showed no visible, moderate, or high GUS activity in the roots, respectively. Similarly, we measured the expression levels of *VIM2*, *CMT3*, and *DRM2* in the shoots of two-week-old wild-type Col-0 plants using RT-qPCR. *VIM2* showed the lowest expression level followed by higher expression of *CMT3* (7.08 fold), and *DRM2* (21.24 fold) (Supplementary Figure S3B), confirming the GUS activity of these genes in shoots.

### 2.6. Promoter Activity of DNA Methylation and Demethylation-Related Genes in Roots following Phytohormone Treatments

**Figure 5:**
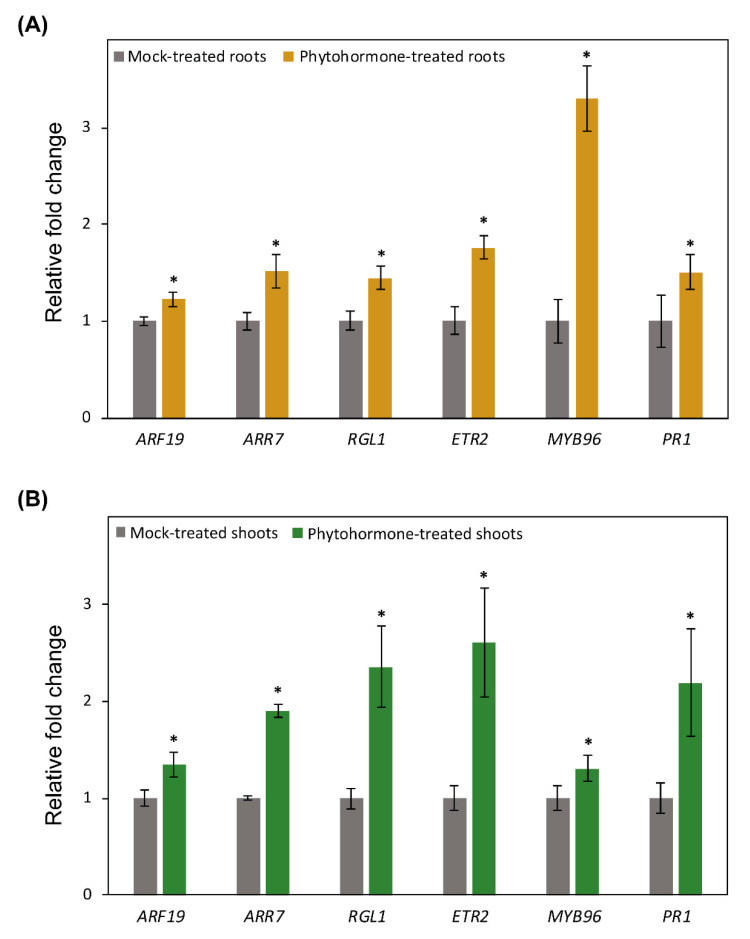
Upregulation of phytohormone marker genes after hormone application. A and B: The expression levels of marker genes for auxin (*ARF19*), cytokinin (*ARR7*), gibberellic acid (*RGL1*), ethylene (*ETR2*), abscisic aid (*MYB96*), and salicylic acid (*PR1*) response were quantified in roots (**A**) and shoots (**B**) of treated and control wild-type Col-0 plants 24 h after hormone application. Relative fold change values were calculated from three biological samples and represent expression in hormone-treated plants relative to mock-treated plants. *Actin8* and *PP2AA3* were used as internal control to normalize gene expression values. Average CT (cycle threshold) values of *Actin8* and *PP2AA3* were used to calculated ^ΔΔ^CT values and gene expression levels. Asterisks indicate statistically significant differences between hormone-treated and mock-treated plants at *p* < 0.05 according to *t*-test.

We next assessed GUS activity in the roots and shoots of 14-day-old transgenic lines growing on MS medium in response to phytohormone treatments, including auxin (AUX), cytokinin (CK), gibberellic acid (GA), ethylene (ET), abscisic acid (ABA), and salicylic acid (SA). The effectiveness of phytohormone treatments in triggering local and systemic responses in leaves and roots was examined by quantifying the expression levels of marker genes for AUX (*auxin response factor19, ARF19*), CK (*Arabidopsis response regulator 7, ARR7*), GA (RGL1), ET (*ethylene response2, ETR2*), ABA (*MYB96*), and SA (*pathogenesis-related gene1, PR1*) responses [66,67,68] in phytohormone-treated plants and the corresponding controls 24 h after phytohormone application using RT-qPCR. Data from three biological replicates revealed significant induction of all these marker genes, both in shoot and root tissues, as compared with the corresponding mock-treated controls (Figure 5A,B), confirming the effectiveness of phytohormone treatments in inducing local and systemic responses. 

**Figure 6:**
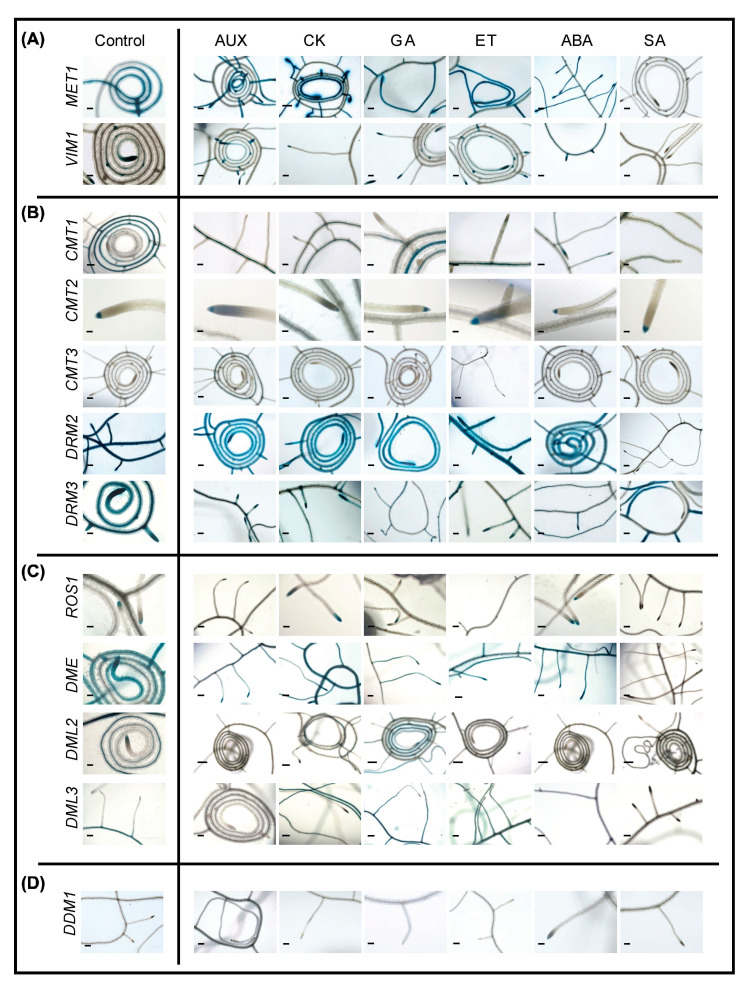
Promoter activity of DNA methylation and demethylation-related genes in two-week-old roots in response to phytohormone treatments. A–C: Histochemical localization of GUS activity directed by promoters of the CG methylation-related genes *MET1* and *VIM1* (**A**), the non-CG and RdDM-related methyltransferases *CMT1*, *CMT2*, *CMT3*, *DRM2*, and *DRM3* (**B**), the demethylases *ROS1*, *DME*, *DML2*, and *DML3* (**C**), and *DDM1* (**D**) in two-week-old roots in response to exogenous application of auxin (AUX), cytokinin (CK), gibberellic acid (GA), ethylene (ET), abscisic acid (ABA), and salicylic acid (SA). Scale bar = 5 mm.

As shown in Figure 6, SA treatments inhibited the expression of *MET1*, *VIM1*, and *DRM2.* With the exception of *DML2*, the expression of the DNA demethylases is also inhibited in response to SA application. Thus, SA seems to inhibit DNA methylation and demethylation-related genes alike (Figure 6). ABA treatment enhanced the expression of *VIM1* but reduced the expression of *DRM3* and *DML3* (Figure 6). AUX treatment induced a reduction in the expression of *DRM2* and *DML3* and slight increase in the expression of *CMT3* (Figure 6B,C). 

Notably, GA treatment elicited an increase in the expression of *DML2* and *DML3* (Figure 6C). CK negatively regulated the expression of *CMT2* in root tips (Figure 6B). The upregulation of *DML2* and *DML3* upon GA treatment and their downregulation following SA treatment points to a possible role of active DNA demethylation in establishing the equilibrium between plant growth and defense responses mediated by GA and SA. The expression patterns of *MET2b*, *MET3*, and *VIM2* did not alter in response to any of the applied hormone treatments (Supplementary Figure S4A). A summary description of changes in the promoter activity of DNA methylation and demethylation-related genes roots in response to various phytohormone treatments is provided in Supplementary Table S2. 

### 2.7. Promoter Activity of DNA Methylation and Demethylation-Related Gene in Shoots following Phytohormone Treatments

**Figure 7:**
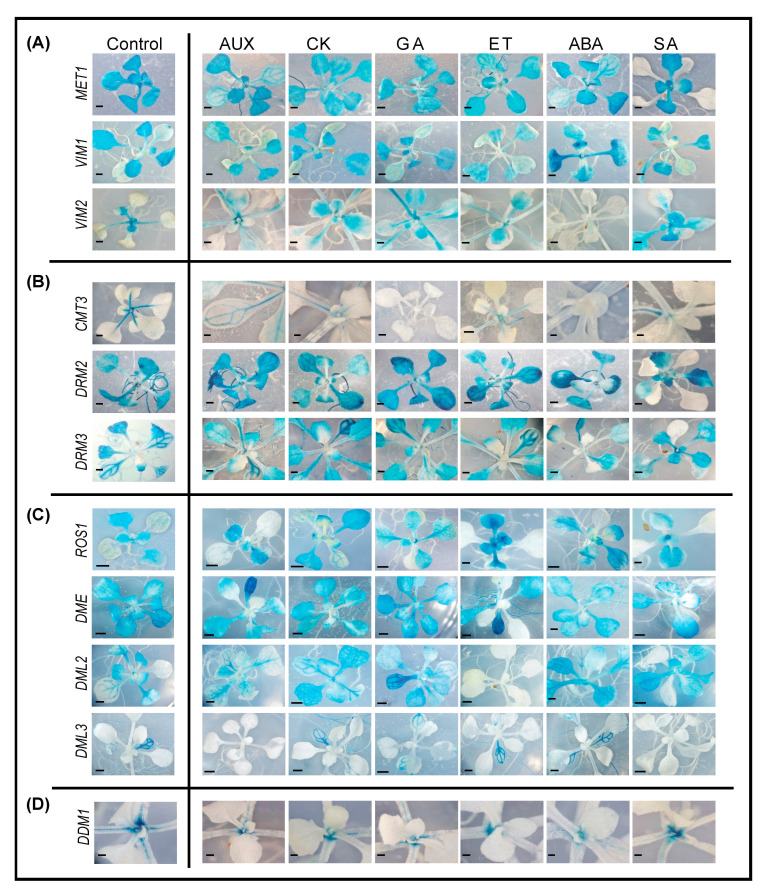
Promoter activity of DNA methylation and demethylation-related genes in two-week-old shoots in response to phytohormone treatments. A–C: Histochemical localization of GUS activity directed by promoters of the CG methylation-related genes *MET1*, *VIM1*, and *VIM2* (**A**), the non-CG and RdDM-related methyltransferases *CMT3*, *DRM2*, and *DRM3* (**B**), and the demethylases *ROS1*, *DME*, *DML2*, and *DML3* (**C**), and *DDM1* (**D**) in two-week-old shoots in response to exogenous application of auxin (AUX), cytokinin (CK), gibberellic acid (GA), ethylene (ET), abscisic acid (ABA), and salicylic acid (SA). Scale bar = 5 mm.

As shown in Figure 7, the promoter activity of genes involved in DNA methylation and demethylation were profoundly altered in phytohormone-treated shoots. Exogenous applications of AUX, CK, GA, ET, and ABA inhibited the expression of *MET1*, *VIM2*, and *DDM1* to various degrees. Notably, GA treatment positively regulated *VIM2* (Figure 7A,D). ABA treatment upregulated *VIM1*, particularly in the cotyledons, and completely suppressed *VIM2* in true leaves (Figure 7A). The expression patterns observed for *VIM1* and *VIM2* in response to ABA and GA treatments suggest that they may play complementary roles in CG methylation, both during development and in response to stress. Overall, genes involved in CG methylation are largely negatively regulated by the phytohormones tested (Figure 7A).

SA treatment negatively impacted *MET1* and *DDM1* expression, but positively influenced *VIM2* expression in leaves (Figure 7A,D). SA and ABA treatments altered the promoter activity of *DRM2* and *DRM3* in emerging leaves (Figure 7B). In addition to its total suppression of *VIM2*, ABA treatment also inhibited the expression of *CMT3*, suggesting that ABA also regulates components of DNA methylation in two-week old shoots (Figure 7A,B). Furthermore, GA suppressed the expression of *CMT3*, but enhanced expression of *DRM2*, giving opposite and complementary expression patterns for these two genes following GA treatment (Figure 7B). 

Both ET and AUX treatments reduced *DME* expression in fast-growing tissues (Figure 7C). Interestingly, the expression of *DML2* or *DML3* was decreased by ET or AUX, respectively (Figure 7C). In response to ET or AUX treatments, *ROS1* appears to be the most responsive DNA demethylase in leaf primordia and young leaves, showing strong GUS activity in these tissues (Figure 7C). SA treatment also impacted DNA demethylation. *DML3* and *DME* expression was suppressed by SA. In contrast, the expression of *ROS1* and *DML2* was slightly enhanced in young leaves by SA treatment (Figure 7C). Thus, in general, SA treatment not only inhibits DNA methylation in older leaves, but it also enhances DNA demethylation in fast-growing tissues. The expression patterns of *MET2b*, *MET3*, and *CMT1* did not alter in response to any of the applied hormone treatments (Supplementary Figure S4B). A summary description of changes in the promoter activity of DNA methylation and demethylation-related genes in response to various phytohormone treatments is provided in Supplementary Table S3.

### 2.8. RT-qPCR Data Validate Gene Expression Changes Induced by Phytohormone Treatments

To validate the gene expression changes of DNA methylation-related genes induced by phytohormone treatments, we used the same RNA samples described in Figure 5 to quantify the expression of genes showing different magnitudes of GUS activity in response to hormone treatments. This includes *VIM1* and *CMT3* in the root tissues of two-week-old wild-type Col-0 plants upon ABA and AUX application, respectively, as well as *DRM2* in the GA-treated roots. The RT-qPCR data of three biological samples revealed two-fold upregulation of *VIM1* in ABA-treated roots as compared with mock-treated control roots (Supplementary Figure S5A)). *CMT3* expression was increased 7.93-fold in AUX-treated roots as compared with mock-treated roots (Supplementary Figure S5B). Similarly, we quantified the expression of *DRM2* in the shoots of two-week-old wild-type Col-0 plants upon GA treatment. The expression levels of *DRM2* was increased 3.14-fold in GA-treated shoots as compared with mock-treated shoots (Supplementary Figure S5C). Together, these data support our conclusion that phytohormone treatments induce gene expression changes of DNA methylation-related genes both in roots and shoots. 

**Figure 8:**
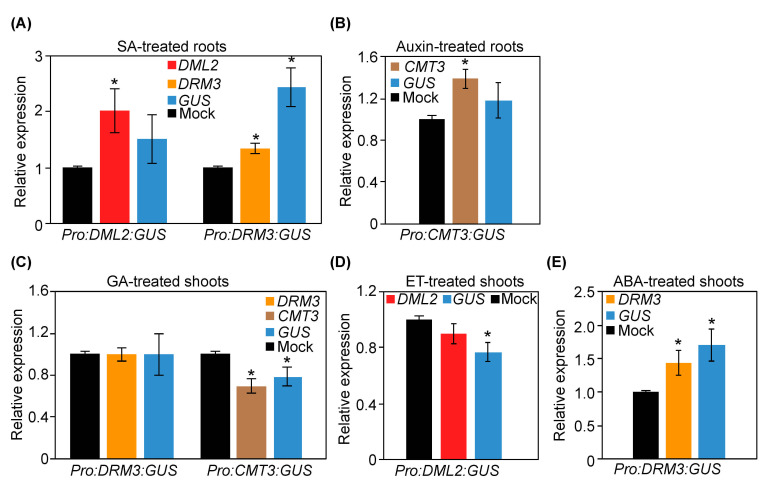
RT-qPCR quantification of *GUS*, *DML2*, *DRM3*, and *CMT3* in the transgenic GUS reporter lines *Pro:DML2:GUS*, *Pro:DRM3:GUS*, and *Pro:CMT3:GUS* in response to various phytohormone treatments. (**A**): Expression levels of *GUS*, *DML2*, and *DRM3* in the roots of two-week-old *Pro:DML2:GUS* or *Pro:DRM3:GUS* plants after SA treatment. (**B**): Expression levels of *GUS* and *CMT3* in the roots of two-week-old *Pro:CMT3:GUS* plants after auxin treatment. (**C**): Expression levels of *GUS*, *DRM3*, and *CMT3* in the shoots of two-week-old *Pro:DRM3:GUS* or *Pro:CMT3:GUS* plants after GA treatment. (**D**): Expression levels of *GUS* and *DML2* in the shoots of two-week-old *Pro:DML2:GUS* plants after ET treatment. (**E**): Expression levels of *GUS* and *DRM3* in the shoots of two-week-old *Pro:DRM3:GUS* plants after ABA treatment. Relative fold change values were calculated from three biological samples and represent expression in roots or shoots of hormone-treated transgenic plants relative to the corresponding tissues of mock-treated transgenic plants. *Actin8* and *PP2AA3* were used as internal controls to normalize gene expression values. Average CT (cycle threshold) values of *Actin8* and *PP2AA3* were used to calculated ^ΔΔ^CT values and gene expression levels. Asterisks indicate statistically significant differences between hormone-treated and mock-treated plants at *p* < 0.05 according to *t*-test.

We next treated 13-day-old plants of the transgenic GUS reporter lines *Pro:DML2:GUS*, *Pro:DRM3:GUS*, and *Pro:CMT3:GUS* with various phytohormone treatments and collected three biological samples of root and shoot tissues 24 h after application. The expression of *GUS* and the corresponding endogenous genes were quantified in roots or shoots relative to the corresponding mock-treated transgenic plants. These reporter lines were selected because they showed increased, decreased, or no change in GUS activity in response to various hormone applications. *GUS* expression in the roots of *DML2* and *DRM3* promoter lines treated with SA revealed upregulation of 1.51- and 2.45-fold compared with the corresponding mock-treated plants (Figure 8A). *DML2* and *DRM3* expression in these samples showed the same trend of upregulation showing 2.0- and 1.34-fold upregulation (Figure 8A). Similarly, the expression of both *GUS* and *CMT3* showed slight upregulation in the auxin-treated roots of *Pro:CMT3:GUS* plants (Figure 8B). 

No notable change in the expression of *GUS* and *DRM3* was detected in the GA-treated shoots of *Pro:DRM3:GUS* plants (Figure 8C). In contrast, both *GUS* and *CMT3* showed comparable downregulation in GA treated shoots of *Pro:CMT3:GUS* plants in comparison with mock-treated shoots (Figure 8C). *GUS* and *DML2* were similarly downregulated in the shoots of *Pro:DML2:GUS* plants treated with ET, showing 0.77- and 0.90-fold, relative to mock-treated shoots (Figure 8D). The expression levels of *DRM3* and *GUS* were induced 1.43- and 1.70-fold, respectively, in the shoots of *Pro:DRM3:GUS* plants compared with the mock-treated shoots (Figure 8E). Together, the gene expression data confirm the accuracy of the transgenic GUS reporter lines in reflecting the transcriptional responses of DNA methylation and demethylation-related genes to various hormone treatments both in shoots and roots.

## 3. Discussion

### 3.1. Promoter Activity of DNA Methylation and Demethylation-Related Genes in Various Plant Tissues and Organs 

Our analysis revealed that DNA demethylases were generally more highly expressed at the mature end of the root, whereas genes involved in DNA methylation were more highly expressed in the newly grown regions of the roots (Figure 1). The spatial expression patterns of our reporter lines, together with the broader distinction between mature and newly grown parts of the root, are congruent with previous DNA methylation profiles reported in root tissues [69]. Our results also substantiate previous conclusions that DNA methylation is important for meristem differentiation and development of fast-growing tissues [60]. In addition, high expression levels of non-CG methyltransferases *DRM2* and *DRM3* at the root tip (Figure 1B) is consistent with the findings that hypermethylation in root tips occurs primarily in the CHH context [69]. High activity of *DRM2* promoter was also reported in root tips [61]. Our finding that several of the tested genes exhibit differential expression patterns in cotyledons, leaf primordia, and young and old leaves suggests a role of DNA methylation in shoot differentiation. This is consistent with the finding that DNA methylation plays a key role in cell identity [55]. 

Transition from juvenile to adult tissues and subsequent reproductive development is highly dependent on DNA methylation, alongside other regulatory factors and exogenous signals [54,70,71]. It has been shown that pre-anthesis buds are hypomethylated directly following the vegetative to reproductive transition, but the mechanism behind this loss of DNA methylation has not been determined [71]. The absence of DNA methyltransferase and demethylase expression in pre-anthesis buds (Figure 3) supports the notion that the hypomethylation of pre-anthesis floral organs is due to passive DNA demethylation. In contrast, reproductive tissues are hypermethylated during floral establishment and in subsequent stages of development [69,71]. Our results point to MET1, VIM1, DRM2, and DRM3 as key contributors to modulating the methylome status for the initial hypermethylation in early floral development (Figure 3A,B). Strong promoter activity of *MET1* and *DRM2* was also reported in Arabidopsis floral organ [61]. In addition, *VIM2* and *DDM1* appear to play a role in later stages of flower development at the receptacle (Figure 3A,D). Our analysis also suggests that methylation status in various floral organs is under tight equilibrium through the activity of genes involved in DNA methylation and demethylation. For example, the high expression of *DME* in carpel, stamen, and pollen was accompanied by high expression of *MET1*, *DRM2*, and *DRM3* (Figure 3A–C).

The strong promoter activity of several genes involved in DNA methylation and demethylation during various stages of seed development implies that the methylomes of developing seeds are established through coordinated functions of methyltransferases and demethylases. *MET2b* and *MET3* were highly expressed during embryogenesis, despite their expression being barely detectable in other tissues (Figure 4A). *MET1* homologs have been reported to express exclusively during embryogenesis [62,72]. This finding suggests that *MET2b* and *MET3* acquired tissue-specific function to support the activity of *MET1* in maintaining CG methylation during seed development. The embryo-specific expression of *VIM1* suggests a coordinated function with MET1 to maintain CG methylation at specific loci, as suggested by Kim et al. [25].

The promoter activity of *CMT1*, *CMT2*, and *DRM2* during embryogenesis is consistent with the programmed increase in CHH methylation during seed development reported in Arabidopsis and other plants [62,73,74,75,76]. This points to their implication in maintenance and de novo CHH methylation, respectively. In this context, it may be important to mention that a role of DRM2 in the maintenance of non-CG sites during embryogenesis can be expected. In addition, the expression of *CMT2* is in line with the significant increase of global CHH-context methylation within TEs located in pericentromeric region during seed development [76]. 

During embryogenesis and seed development, the endosperm tissues are hypomethylated, particularly at short TEs located nearby genes [73,77]. *DME* was established as the main demethylase responsible for genome-wide hypomethylation of Arabidopsis endosperm [73,77,78]. In addition to the role of *DME*, our data suggest a key role for *DML2* and *DML3* in this process, and their role in establishing a hypomethylated endosperm merits further analysis (Figure 4C). 

Tomato DML2-mediated DNA demethylation contributes to global hypomethylation during fruit ripening [48,79,80,81]. Our assays show that *ROS1* and/or *DME* were expressed at silique internodes throughout development and that *DME* is highly expressed in the replum and septum (Figure 3C), suggesting their implication in silique maturation. Together with the expression of genes involved in DNA methylation in internodes, replum, stigma, and style (Figure 3A,B), our results suggest that DNA methylation and demethylation have a role in silique development and fruit ripening, as recently reported [12,80,82,83].

### 3.2. Phytohormone-Induced Changes in the Promoter Activity of DNA Methylation and Demethylation-Related Genes

Our results provide evidence that phytohormones can alter DNA methylation dynamics in a specific and coordinated manner. In root tissues, we found that SA treatment inhibited the promoter activity of several genes but also activated the expression of *CMT1*, *DRM3*, and *DML2* (Figure 6 and Figure 8A). The role of SA in root initiation and root growth [84] may be brought about in part through changes of DNA methylation pathways. SA-induced *CMT1* expression in lateral roots implies a unique function of this enzyme in salicylic acid-mediated methylome changes in lateral roots (Figure 6B). Consistent with the role of SA in RAM patterning via auxin redistribution [85], our results point to a possible role of SA-dependent induction of *CMT2* in this process, because *CMT2* was the only gene expressed in the RAM of both AUX- and SA-treated roots (Figure 6B). 

MET1 and the RdDM pathway have been identified to regulate active DNA demethylation through the methylation of a regulatory sequence in the *ROS1* promoter [41]. Our GUS assays in roots and shoots support this regulation and implicate SA in this regulatory mechanism. In roots, the promoter activity of *MET1*, *DRM2*, and *DRM3* in root tips, and *ROS1* in root cap, was strongly repressed in response to SA treatment (Figure 6). Similarly, in shoots, SA treatment reduced or eliminated the expression of *MET1*, *DRM2*, *DRM3*, and *ROS1* in older leaves and/or cotyledons (Figure 7). ABA may also be involved in this regulatory mechanism, particularly in younger leaves, as the expression of *MET1* and *ROS1* was reduced or eliminated in these tissues upon ABA application (Figure 7). 

AUX, CK, ET and ABA treatments also impacted the promoter activity of certain DNA methylation and demethylation-related genes in various root tissues, suggesting that a hormone-linked methylome regulation mechanism may contribute to root growth and development. In many cases, phytohormone treatments resulted in an obvious impact on *GUS* expression in the shoots. Some genes showed similar responses to various hormone treatments, as in the case of *DDM1*, whose expression was inhibited in response to all hormone applications in shoots (Figure 7D). Other genes showed opposite responses to certain hormone treatments. For example, *VIM2* was positively regulated by SA and GA treatments in older leaves, but its expression was strongly suppressed by ABA (Figure 7A). Interestingly, *ROS1* expression is enhanced in response to GA and ET treatments, but repressed by CK (Figure 7C), suggesting that ROS1-mediated DNA demethylation can be modulated by phytohormone signaling. Additionally, some hormone applications can differentially influence the expression of gene family members. For instance, *VIM1* was upregulated in leaves following ABA treatment, whereas *VIM2* expression was almost completely abolished (Figure 7A). We also realized that hormone applications triggered tissue-specific expression patterns. In this context, we found that *DRM3* was upregulated in younger leaves and downregulated in older leaves in response to SA treatment (Figure 7B). Additionally, *MET1* expression is reduced following ABA treatment in shoots, but not in roots (Figure 6A and Figure 7A), supporting previous gene expression data showing downregulation of *MET1* in a concentration-dependent manner in response to ABA treatments [86]. Taken together, these findings suggest that hormone signaling may contribute to the dynamic changes of DNA methylation associated with tissues development. 

## 4. Materials and Methods

### 4.1. Plant Materials and Growth Conditions

All transgenic Arabidopsis lines were generated in the Col-0 ecotype background. Plants were gown in controlled growth chambers at 22 °C under long day conditions, 16 h of light (75 µmol m^−2^ s^−1^) and 8 h of dark. 

### 4.2. Generation of GUS Reporter Lines

Promoter regions (up to 2 kb region upstream of the translation start codon ATG) were amplified from Col-0 genomic DNA using primers containing restriction enzyme sites (Supplementary Table S1). In some cases, the distance between the ATG and the 3′untranslated region (3′UTR) of the neighboring gene is less than 2 kb. Therefore, we used the largest possible promoter region without including the 3′UTR of the adjacent genes. Amplified PCR products were digested, purified, and cloned upstream of the β-glucuronidase (GUS) gene in the binary vector pBI101. Constructs were confirmed by sequencing before transferring into *Agrobacterium tumefaciens* (strain C58). The functionality of these constructs was validated using an *A. tumefaciens* infiltration assay [87]. Four-day-old seedlings were infiltrated with transformed *A. tumefaciens* for 36 h, and seedlings were analyzed for GUS activity. Within 0.5 to 24 h of staining, colorimetric changes were visually observed for all infiltrated seedlings, confirming the function of all gene promoters. 

Agrobacteria containing the binary vectors were then used to transform Arabidopsis (Col-0) using the floral dip method [88]. Transgenic T1 plants were screened on MS medium containing 50 mg/L kanamycin and 100 mg/L cefotaxime. Transgenic T1 lines were self-fertilized to generate T2 lines. T2 and subsequent generations were used for GUS assays.

### 4.3. Histochemical Analysis of GUS Activity

At least two independent transgenic lines were assayed for promoter activity, with at least six biological replicates. Seeds were grown in 12-well plates containing MS medium, and 14-day-old plants were assayed for GUS activity in root and shoot tissues. For GUS assays in reproductive tissues, transgenic plants were grown in soil at 22 °C under long day conditions, and reproductive tissues, including buds, open flowers, emerging siliques, fully developed siliques, and mature siliques were assayed for GUS activity [89] using the X-Gluc substrate (5-bromo-4-chloro-3-indolyl-beta-D-glucuronic acid, cyclohexylammonium salt) (Rose Scientific, Cincinnati, OH, USA) at 37 °C and checked every 30 min for colorimetric changes. The X-Gluc substrate was dissolved in 0.1 M sodium phosphate buffer (pH = 7) containing 20% methanol, 0.3% Triton X-100, 0.5 mM potassium ferricyanide, and 0.5 mM potassium ferrocyanide at a concentration of 1 mg/mL. GUS staining time ranged from 30 min to 12 h based on the promoter activity. Once the staining was clearly visible, the reaction was terminated, to avoid overstaining, by replacing the GUS solution with 70% ethanol. The samples were then stored in ethanol at room temperature. Developing seeds were also precleared using 1:1 Acetic acid ethanol for 4–6 h and then cleared for 5 days using 75% strength Hoyer’s medium without gum arabic [90].

### 4.4. Phytohormone Treatments

Seeds of the transgenic GUS reporter lines were surface-sterilized and planted in 12-well plates containing MS medium in controlled growth chambers at 22 °C and 16 h light/8 h dark cycle. At 13 days after planting, phytohormone and control treatments were applied to the transgenic seedlings. Excess liquids were removed using autoclaved filter papers. Phytohormone and control treatments were maintained for 24 h before determining GUS activity, both in roots and shoots, by adding GUS solution directly to the 12-well plates. The following phytohormones were used at the designated concentrations: 5 nM Indole-3-Acetic Acid (IAA, AUX), 15 nM 6-Benzylaminopurine (BAP, CK), 75 µM gibberellic acid (GA), 10 µM 1-aminocyclopropane-1-carboxylic acid (ACC, ET), 10 µM abscisic acid (ABA), and 2 mM salicylic acid (SA), as previously described [91,92,93,94,95]. Stock solutions of these phytohormones were prepared in 100% ethyl alcohol (IAA, ABA, and SA), 1 N NaOH (BAP), or distilled water (GA and ACC). Mock treatments for control plants were carried out using distilled water (for GA and ACC treatments), 1.63 × 10^−9^ mol/L, 0.224 mol/L, and 0.08003 mol/L ethyl alcohol for IAA, ABA, and SA treatments, respectively. Mock-treated control plants were included for each experiment and compared to treated plants. Phytohormone treatments were performed with two independent transgenic lines per construct.

### 4.5. RT-qPCR Analysis

The expression levels of phytohormone marker genes for auxin (*auxin response factor19* (*ARF19*), *AT1G19220*), cytokinin (*arabidopsis response regulator7* (*ARR7*), *AT1G19050*), gibberellic acid (*RGA-like 1* (*RGL1*)), ethylene (*ethylene response2* (*ETR2*), *AT3G23150*), abscisic aid (*MYB96*, *AT3G22830*), and salicylic acid (*pathogenesis-related gene1* (*PR1*), *AT2G14610*) were quantified in phytohormone-treated roots and shoots. The expression levels of *VIM2*, *CMT3*, *DRM2*, and *DRM3* were measured in untreated roots and/or shoots of wild-type Col-0 plants. The expression levels of *VIM1*, *CMT3*, and *DRM2* were also quantified in wild-type Col-0 plants in response to ABA, AUX, and GA applications, respectively. Additionally, the transgenic GUS reporter lines *Pro:DML2:GUS*, *Pro:DRM2:GUS*, and *Pro:CMT3:GUS* were used to quantify the expression of *GUS* and the corresponding endogenous genes in root or shoot tissues after various hormone treatment. Seeds of Arabidopsis wild-type ecotype Col-0 were planted in 12-well plates containing MS medium, and 13-day-old seedlings were subjected to various phytohormone and control treatments as described above. Twenty-four hours after the treatments, leaf and root tissues were collected in three biological samples and used for RNA extraction and RT-qPCR quantification. Total RNA was extracted using the Zymo Direct-zol RNA mimiprep kit (R2050) (Zymo Research, Irvine, CA, USA). RT-qPCR quantification was performed using 30 ng of DNase-treated RNA per reaction with Verso SYBR green 1-step RT-qPCR (Thermo Scientific, Waltham, MA, USA), according to the manufacturer’s instructions. RT-qPCR was performed in the QuantStudio 6 Flex Real-Time PCR System (Applied Biosystems, Foster City, CA, USA) using the following program: 50 °C for 15 min, 95 °C for 15 min followed by 40 cycles of 95 °C for 15 s, 60 °C for 90 s, and 72 °C for 30 s. The PCR products were then subjected to 95 °C for 15 s and 60 °C for 75 s, followed by a slow gradient from 60 °C to 95 °C to generate the dissociation curves. Gene expression levels were normalized using two different reference genes: *Actin8* (*AT1G49240*) and *PROTEIN PHOSPHATASE 2A SUBUNIT A3* (*PP2AA3*, *AT1G13320*) [96]. Phytohormone-treated roots and shoots were then compared to mock-treated roots and shoots, respectively. Primer sequences for RT-qPCR assays are provided in Supplementary Table S1.

### 4.6. Light Microscopy and Imaging 

Roots were imaged immediately because of their rapid degradation, even preserved in ethanol. Images of media-grown roots were captured from the bottom of the plate using an EVOS M7000 microscope. Shoots and reproductive tissues, on the other hand, were incubated in 70% ethanol for a few days to remove chlorophyll pigmentation, allowing GUS expression patterns to be clearly visible. These tissues were then imaged using a Zeiss Stemi 2000-CS dissecting microscope.

### 4.7. RNA-Seq Data Analysis 

Gene expression data of DNA methylation and demethylation-related genes in roots, root tips, leaf, petiole, buds, open flower, and mature silique were downloaded from TraVA (Transcriptome Variation Analysis, http://travadb.org/, accessed on 22 September 2020) [65]. Absolute RNA-seq read counts normalized by trimmed mean of M samples (TMM) were log10 transformed and used to generate a heatmap using the R package gplots and heatmap.2 function.

## Data Availability

Not applicable.

## Supplementary Materials

The following are available online at https://www.mdpi.com/article/10.3390/ijms22189681/s1. Supplementary Figure S1: GUS staining assays of transgenic reporter lines showing absence of expression of the indicated DNA methylation and demethylation-related genes in roots (A), shoots (B), floral tissues and organ (C), and various stages of embryogenesis (D). Scale bar = 5 mm, Supplementary Figure S2: Heatmap representation of gene expression levels of DNA methylation and demethylation-related genes across different tissues and organs, Supplementary Figure S3: RT-qPCR quantification of selected DNA methylation-related genes in 2-week-old wild-type Col-0 plants. A: Expression levels of *VIM2*, *CMT3*, and *DRM3* in the roots of 2-week-old wild-type Col-0 plants. B. Expression levels of *VIM2*, *CMT3*, and *DRM2* in the shoots of 2-week-old wild-type Col-0 plants. Relative fold change values were calculated from three biological samples and represent expression levels of *CMT3*, *DRM2*, or *DRM3* relative to *VIM2*, which were set to 1. *Actin8* and *PP2AA3* were used as internal control to normalize gene expression values. Average CT (cycle threshold) values of *Actin8* and *PP2AA3* were used to calculated ^ΔΔ^CT values and gene expression levels. Asterisks indicate statistically significant differences from the expression values of *VIM2* at *p* < 0.05 according to *t*-test, Supplementary Figure S4: GUS staining assays of transgenic reporter lines showing no changes in expression of the indicated DNA methylation-related genes in roots (A), or shoots (B) following treatment with (from left to right) auxin (AUX), cytokinin (CK), gibberellic acid (GA), ethylene (ET), abscisic acid (ABA), or salicylic acid (SA). Scale bar = 5 mm, Supplementary Figure S5: RT-qPCR quantification of selected DNA methylation-related genes in two-week-old wild-type Col-0 plants in response to the indicated phytohormone treatments. A: Expression levels of *VIM1* in the root tissues of two-week-old wild-type Col-0 plants after ABA application. B: Expression levels of *CMT3* in the root tissues of two-week-old wild-type Col-0 plants after AUX application. C: Expression levels of *DRM2* in shoots of two-week-old wild-type Col-0 plants after GA application. Relative fold change values were calculated from three biological samples and represent expression in roots or shoots of hormone-treated plants relative to the corresponding tissues of mock-treated plants. *Actin8* and *PP2AA3* were used as internal control to normalize gene expression values. The average CT (cycle threshold) values of *Actin8* and *PP2AA3* were used to calculated ^ΔΔ^CT values and gene expression levels. Asterisks indicate statistically significant differences between hormone-treated and mock-treated plants at *p* < 0.05 according to *t*-test, Supplementary Table S1: Gene identification numbers and primers used in this study, Supplementary Table S2: Summary description of changes in promoter activity of DNA methylation and demethylation-related genes in two-week-old roots in response to phytohormone treatments, Supplementary Table S3: Summary description of changes in promoter activity of DNA methylation and demethylation-related genes in two-week-old shoots in response to phytohormone treatments.

## Abbreviations

ABA	Abscisic acid
AUX	Auxin
CK	Cytokinin
ET	Ethylene
GA	Gibberellic acid
RdDM	RNA-directed DNA methylation
SA	Salicylic acid
RAM	Root apical meristem
