# Supplementary material for: Expression Patterns of DNA Methylation and Demethylation Genes during Plant Development and in Response to Phytohormones
Source: Int J Mol Sci. 2021 Sep 7;22(18):9681. doi: 10.3390/ijms22189681 (PMC8470644; doi:10.3390/ijms22189681)
Supplement: Supplementary file 1 [file ijms-22-09681-s001.zip › Supplementary Figures.pdf]

Supplementary Figures S1-S5

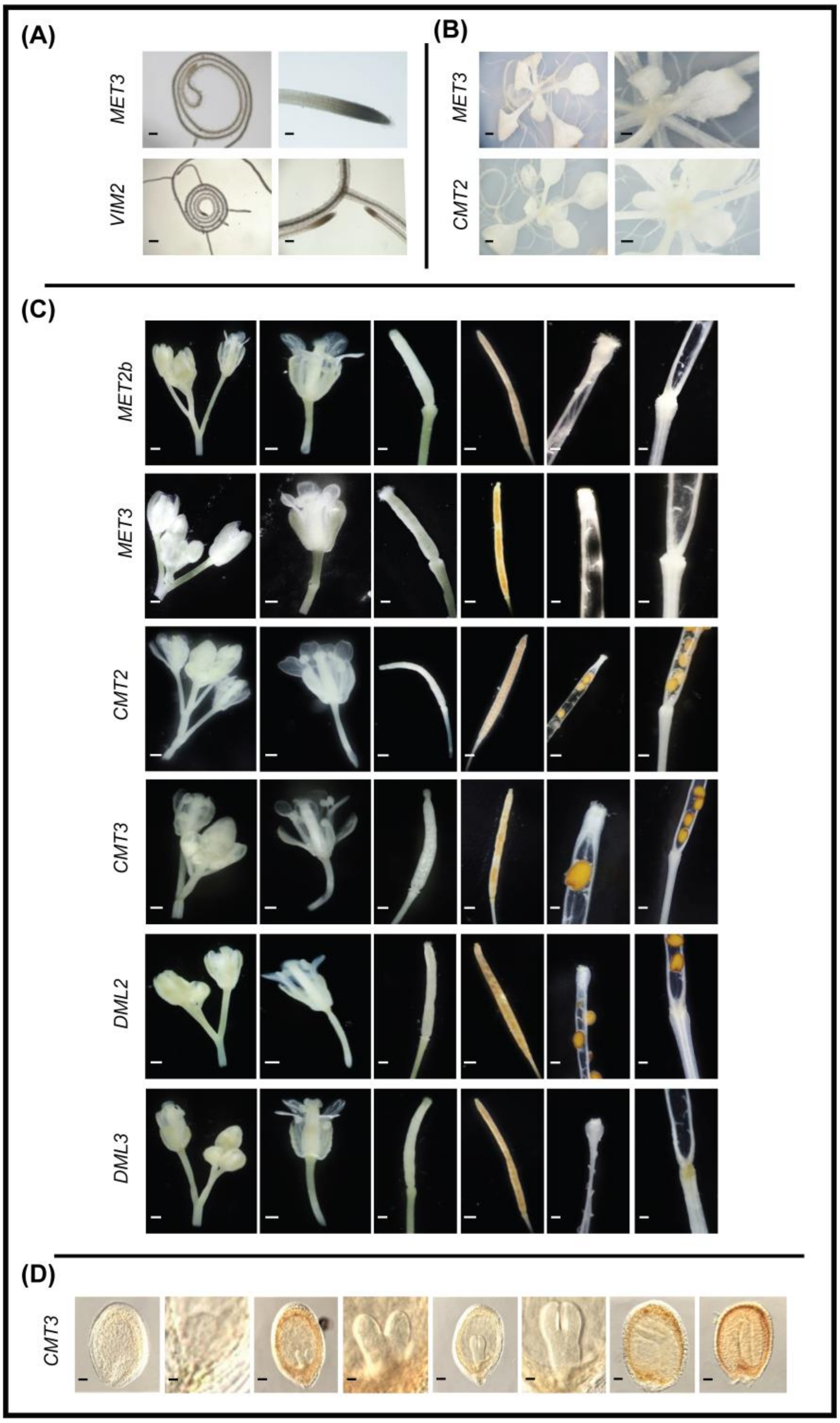

Figure S1. GUS staining assays of transgenic reporter lines showing absence of expression of the indicated DNA methylation and demethylation-related genes in roots (A), shoots (B), floral tissues and organ (C), and various stages of embryogenesis (D). Scale bar = 5 mm.

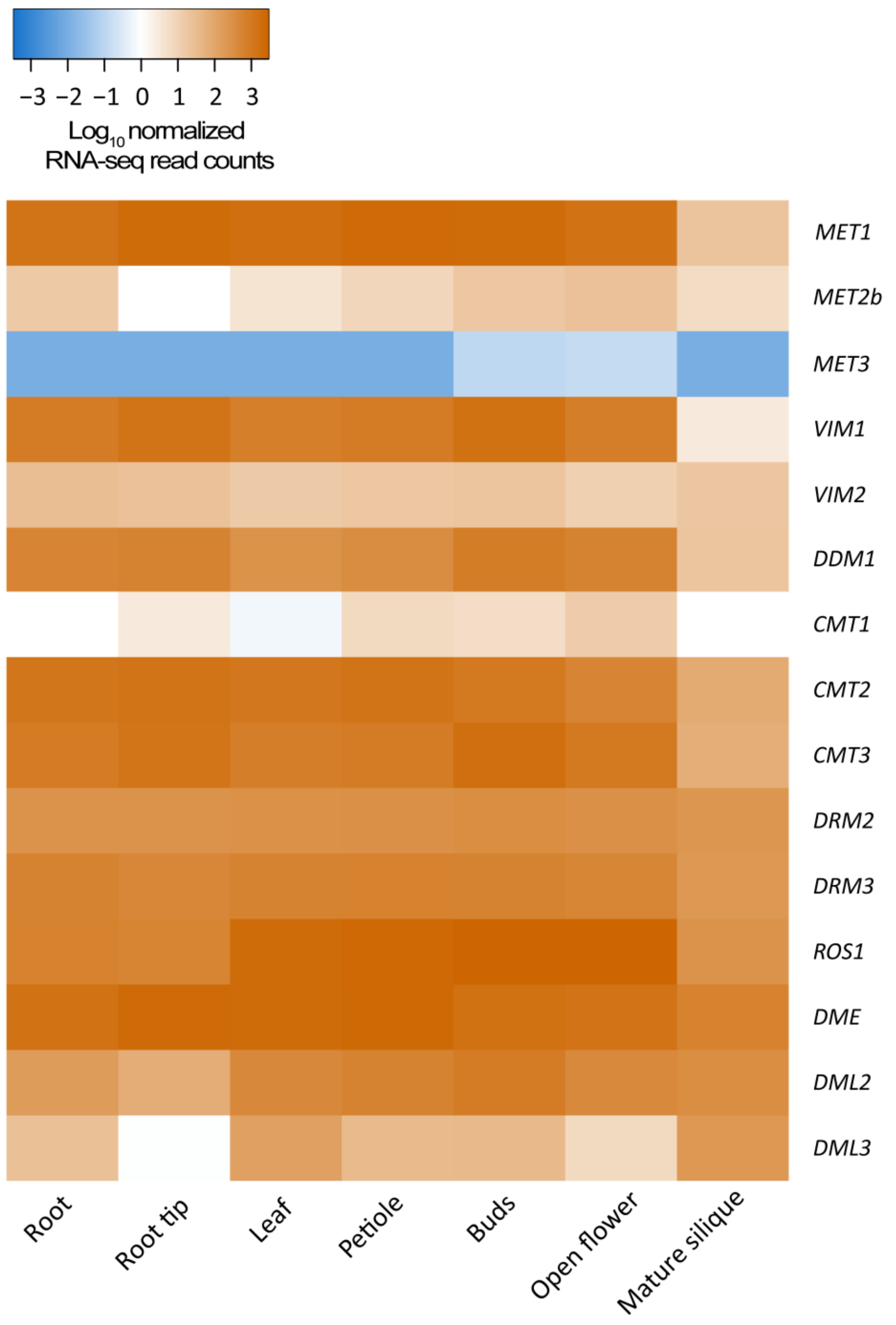

Figure S2. Heatmap representation of gene expression levels of DNA methylation and demethylation-related genes across different tissues and organs.

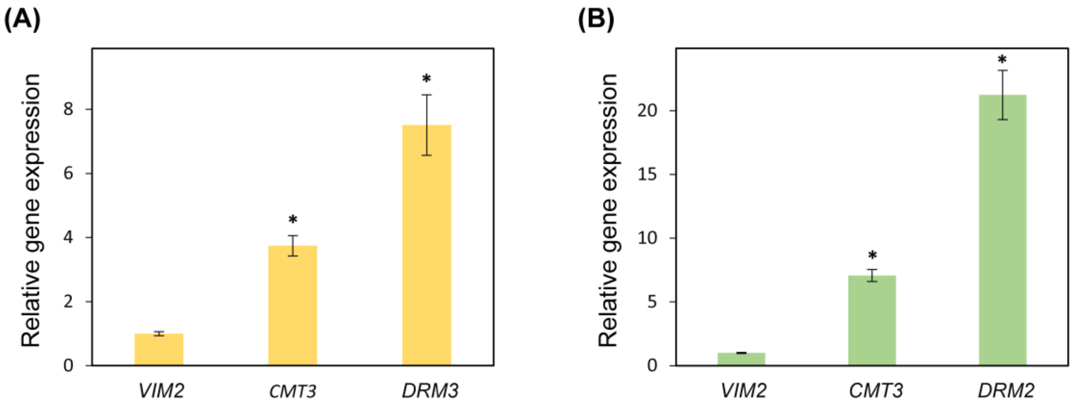

Figure S3. RT-qPCR quantification of selected DNA methylation-related genes in 2-week-old wild-type Col-0 plants. A: Expression levels of *VIM2*, *CMT3*, and *DRM3* in the roots of 2-week-old wild-type Col-0 plants. B. Expression levels of *VIM2*, *CMT3*, and *DRM2* in the shoots of 2-week-old wild-type Col-0 plants. Relative fold change values were calculated from three biological samples and represent expression levels of *CMT3*, *DRM*2, or *DRM3* relative to *VIM2*, which were set to 1. *Actin8* and *PP2AA3* were used as internal control to normalize gene expression values. Average CT (cycle threshold) values of *Actin8* and *PP2AA3* were used to calculated $^{\Delta\Delta}$CT values and gene expression levels. Asterisks indicate statistically significant differences from the expression values of *VIM2* at $p < 0.05$ according to *t*-test.

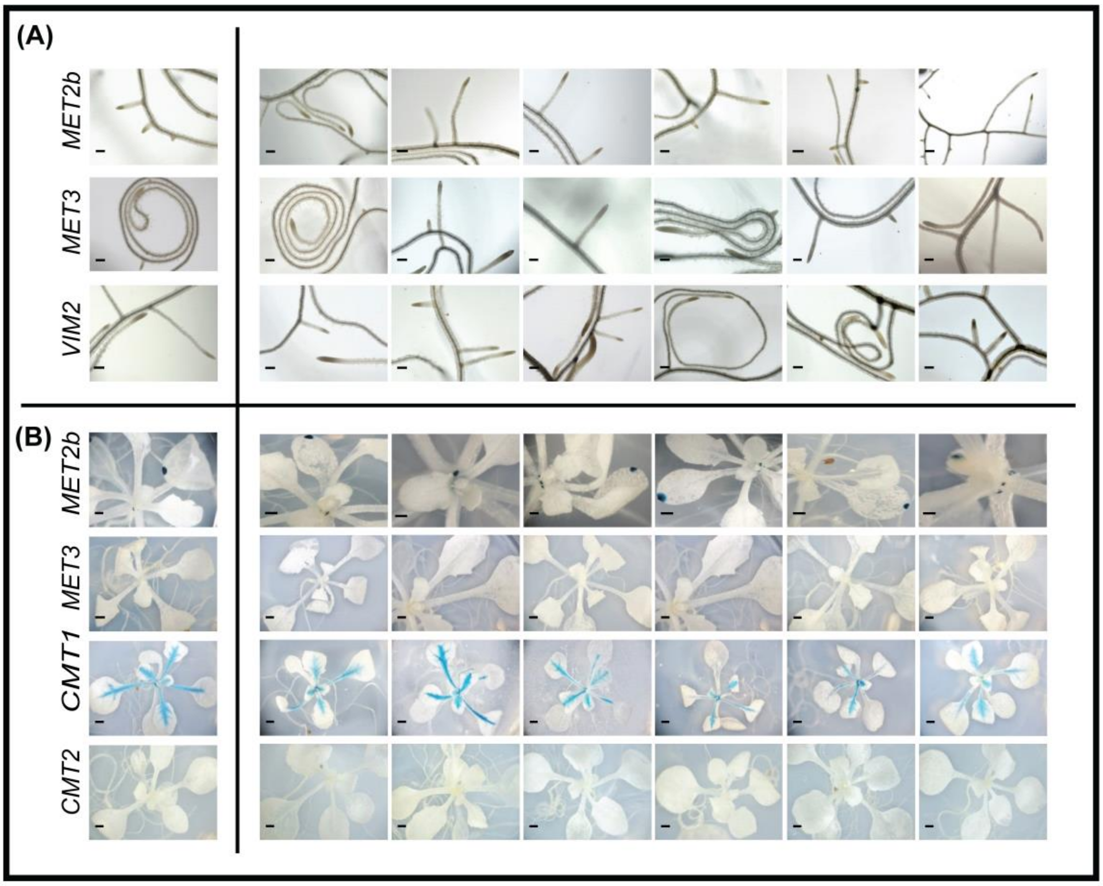

Figure S4. GUS staining assays of transgenic reporter lines showing no changes in expression of the indicated DNA methylation-related genes in roots (A), or shoots (B) following treatment with (from left to right) auxin (AUX), cytokinin (CK), gibberellic acid (GA), ethylene (ET), abscisic acid (ABA), or salicylic acid (SA). Scale bar = 5 mm.

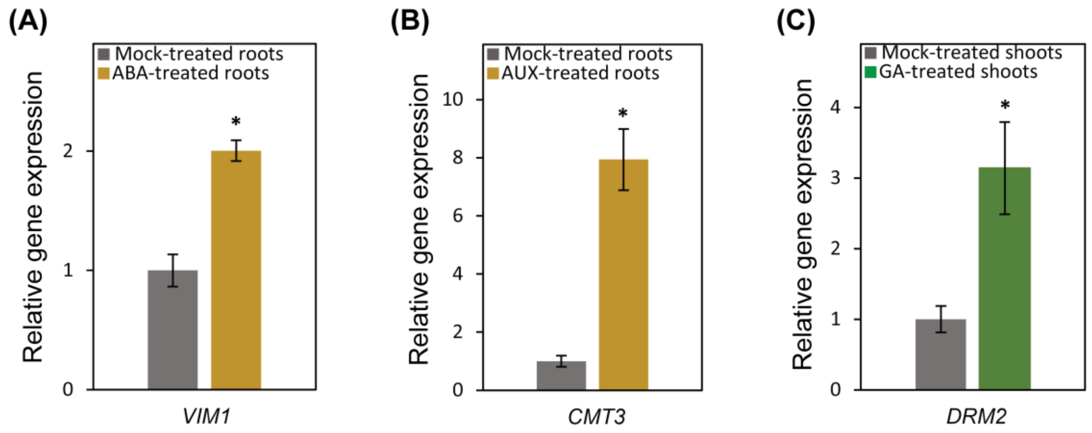

Figure S5. RT-qPCR quantification of selected DNA methylation-related genes in two-week-old wild-type Col-0 plants in response to the indicated phytohormone treatments. A: Expression levels of *VIM*1 in the root tissues of two-week-old wild-type Col-0 plants after ABA application. B: Expression levels of *CMT3* in the root tissues of two-week-old wild-type Col-0 plants after AUX application. C: Expression levels of *DRM2* in shoots of two-week-old wild-type Col-0 plants after GA application. Relative fold change values were calculated from three biological samples and represent expression in roots or shoots of hormone-treated plants relative to the corresponding tissues of mock-treated plants. *Actin8* and *PP2AA3* were used as internal control to normalize gene expression values. The average CT (cycle threshold) values of *Actin8* and *PP2AA3* were used to calculated $^{\Delta\Delta}$CT values and gene expression levels. Asterisks indicate statistically significant differences between hormone-treated and mock-treated plants at $p < 0.05$ according to t-test.
